# Roles and Mechanisms of Dopamine Receptor Signaling in Catecholamine Excess Induced Endothelial Dysfunctions

**DOI:** 10.7150/ijms.96550

**Published:** 2024-07-22

**Authors:** Zhen Yang, Yingrui Li, Mengying Huang, Xin Li, Xuehui Fan, Chen Yan, Zenghui Meng, Bin Liao, Nazha Hamdani, Xiaoli Yang, Xiaobo Zhou, Ibrahim El-Battrawy, Ibrahim Akin

**Affiliations:** 1 Department of Ophthalmology, Affiliated Hospital of North Sichuan Medical College, 637000 Nanchong, Sichuan, China.; 2 First Department of Medicine, Medical Faculty Mannheim, University Medical Centre Mannheim (UMM), Heidelberg University, 68167 Mannheim, Germany.; 3 European Center for AngioScience (ECAS), German Center for Cardiovascular Research (DZHK) partner site Heidelberg/ Mannheim, and Centre for Cardiovascular Acute Medicine Mannheim (ZKAM), Medical Centre Mannheim, Heidelberg University, Germany.; 4 Key Laboratory of Medical Electrophysiology of Ministry of Education and Medical Electrophysiological Key Laboratory of Sichuan Province, Institute of Cardiovascular Research, Southwest Medical University, 646000 Sichuan, China.; 5 Department of Cardiac Macrovascular Surgery, Affiliated Hospital of Southwest Medical University, 646000 Sichuan, China.; 6 Department of Cellular and Translational Physiology, Institute of Physiology, Ruhr University Bochum, 44801 Bochum, Germany and Institut für Forschung und Lehre (IFL), Molecular and Experimental Cardiology, Ruhr University Bochum, 44801 Bochum, Germany.; 7 Bergmannsheil Bochum, Medical Clinic II, Department of Cardiology and Angiology, Ruhr University, Bochum, Germany.

**Keywords:** dopamine receptor, small conductance calcium-activated potassium channel, Takotsubo syndrome, endothelial dysfunction, nitric oxide.

## Abstract

Endothelial dysfunction may contribute to pathogenesis of Takotsubo cardiomyopathy, but mechanism underlying endothelial dysfunction in the setting of catecholamine excess has not been clarified. The study reports that D1/D5 dopamine receptor signaling and small conductance calcium-activated potassium channels contribute to high concentration catecholamine induced endothelial cell dysfunction.

For mimicking catecholamine excess, 100 μM epinephrine (Epi) was used to treat human cardiac microvascular endothelial cells. Patch clamp, FACS, ELISA, PCR, western blot and immunostaining analyses were performed in the study.

Epi enhanced small conductance calcium-activated potassium channel current (I_SK1-3_) without influencing the channel expression and the effect was attenuated by D1/D5 receptor blocker. D1/D5 agonists mimicked the Epi effect, suggesting involvement of D1/D5 receptors in Epi effects. The enhancement of I_SK1-3_ caused by D1/D5 activation involved roles of PKA, ROS and NADPH oxidases. Activation of D1/D5 and SK1-3 channels caused a hyperpolarization, reduced NO production and increased ROS production. The NO reduction was membrane potential independent, while ROS production was increased by the hyperpolarization. ROS (H2O2) suppressed NO production.

The study demonstrates that high concentration catecholamine can activate D1/D5 and SK1-3 channels through NADPH-ROS and PKA signaling and reduce NO production, which may facilitate vasoconstriction in the setting of catecholamine excess.

## Introduction

Takotsubo cardiomyopathy (TTC), also called Takotsubo syndrome or stress cardiomyopathy, is a stress-induced cardiac disorder. TTC can display clinical characters similar to that of acute coronary syndromes (ACS) 1, 2. In TTC-patients, chest pain, dyspnoea, syncope, ST-segment elevation or T-wave inversion in ECG are common 3, 4. However, TTC differs from ACS in the reality that TTC has no coronary artery stenosis and the disease can be reversable within days or weeks if appropriate therapy is applied 5. Apical ballooning, resulting from a regional left ventricular dysfunction (impairment of contraction mainly in apical but not in basal cardiomyocytes), is a typical sign in hearts of TTC-patients 6.

The prevalence of TTC is approximately 1-3% 7, 8 of all and 5-6% of female patients with STEMI (ST-elevation myocardial infarction) 9. Around 90% of TTC patients are female between 67-70 years 10. Women > 55 years have a risk of TTC 10 folds higher than men 11.

Although increasing TTC cases have been reported, the pathogenesis of TTC is still unclear. Up to date, supposed mechanisms include: coronary artery spasm, coronary microvascular dysfunction, toxic effects of catecholamine, etc. 12. It is widely accepted that catecholamine excess is a central pathogenic factor. In most cases, TTC occurs after physical or emotional stresses 5. In addition, the catecholamine level in serum was increased in more than 70% of TTC cases 13. Also, TTC occurred in some patients after injection with epinephrine 14-17. In animal models, injection of high doses of epinephrine caused TTC-like changes in the heart such as regional abnormal contractions and apical ballooning 18. Taken together, catecholamine excess contributes to TTC pathogenesis. Therefore, roles and mechanism of catecholamine excess for the pathogenesis of TTC have been extensively investigated, mainly focused on adrenergic stimulation and ß-adrenoceptor signaling.

Given that TTC is related with catecholamine excess, application of beta-blockers should be a useful strategy for TTC treatment. Surprisingly, application of beta-blocker in the acute phase displayed no beneficial effect on the in-hospital mortality of TTC 19. Similarly, no benefits of beta-blockers were detected in 1 year of follow-up of TTC patients 10. Furthermore, two meta-analyses detected no benefit of beta-blockers in preventing recurrence of TTC 20, 21. These data suggest that non-beta-receptor signaling may also contribute to the pathogenesis of TTC.

Dopamine is a precursor to norepinephrine and contributes to catecholamine effects. Dopamine receptors also exist in the heart including cardiomyocytes and coronary artery, and may play roles for heart function or disease 22-24. In animals, injection of dopamine also induced TTC-like changes in the animal heart 25. In patients, administration of high-dose dopamine induced TTC-like cardiomyopathy 26, 27, but underlying mechanisms were not investigated. Whether dopamine receptor signaling contributes to TTC pathogenesis is not clear.

The ST-segment elevation in ECG in TTC-patients, who have no coronary stenosis, hint at a coronary spasm. A recent study, using animal model, demonstrated experimentally that TTC is a coronary microvascular disease 28. It is well-established that endothelial cells play important roles for controlling coronary artery contraction/relaxation 29. Endothelial cells can release vasoconstrictor like endothelin-1 (ET-1) and vasodilator like nitric oxide (NO), which play an important role in controlling coronary flow 29. Under physiological conditions, the balance of ET-1/ NO regulation warrants normal tone of coronary artery. Injury in endothelium can impair endothelium and ET-1/NO balance, causing endothelial dysfunctions. Endothelial dysfunction, which is characterized by an imbalance between vasoconstriction and vasodilation 30, can contribute to the development of cardiovascular diseases like atherosclerosis 31 and microvascular dysfunctions 32.

Vascular endothelial dysfunctions were observed in a study in TTC-patients 33. That study including 22 TTC patients detected a significant increase in endothelial dysfunction in the patients. Besides, the ET-1 level in plasma of TTC-patients was higher than that in healthy subjects 34. These results suggest that endothelial dysfunction may participate in the development of TTC. However, few studies investigated the roles of endothelial dysfunction in the pathophysiology of TTC.

Ion channels in endothelial cells have been proven to be important for endothelium functions 29, 35, 36. In endothelial cells, small (SK1-3) and intermediate (SK4) conductance calcium activated K^+^ channels are most frequently investigated and well-characterized 35. The arterial endothelium can control vascular tone via releasing vasodilating factors as nitric oxide, prostacyclin and endothelium-derived hyperpolarizing factor (EDHF) 35. Endothelial SK channels are suggested to be involved in EDHF-functions 37. Alteration of EDHF-mediated responses was detected in some diseases such as hypertension, atherosclerosis, hypercholesterolemia, heart failure, diabetes and sepsis 38.

In general, ion channels in endothelial cells are important for endothelial functions. Changes of ion channel functions may cause endothelial dysfunctions. However, whether alteration of SK channel function in endothelial cells contributes to endothelial dysfunction in TTC is unknown. Therefore, we designed this study to investigate endothelial dysfunctions in the setting of TTC, focusing on contributions of dopamine receptor and SK1-3 channels.

## Materials and Methods

The human cardiac microvascular endothelial cells (HCMECs) were purchased from PromoCell (Cat. No.: C-12285, PromoCell GmbH, Heidelberg, Germany). The tissues used by PromoCell for the isolation of human cell cultures originate from donors who have signed a declaration of consent. PromoCell's donor acquisition, documents and cell collection process have been assessed and evaluated and approved by the ethical commission of the doctor's medical association of Baden-Württemberg (#219-04).

Detailed information of methods and materials is supplied in the supplemental data. Shortly, HCMECs were treated with epinephrine (100 μM for 1 h) to mimic the setting of TTC (catecholamine excess). SK1-3 channel currents and cell membrane potential were measured by patch clamp whole recording techniques. Gene and protein expression levels were measured by PCR and western blot as well as immunostaining. NO production was measured by ELISA. ROS production was measured by FACS.

All data are presented as mean ± SEM and were analyzed with SigmaPlot 14.0 (Systat GmbH, Germany). The unpaired t-test was used for comparisons of two independent groups with normal distribution. One-way ANOVA with Holm-Sidak post-test was performed for comparisons of more than two groups. P < 0.05 (two-tailed) was considered significant.

## Results

### Dopamine D1/D5 receptor activation increased SK1-3 currents in HCMECs

**Figure 1:**
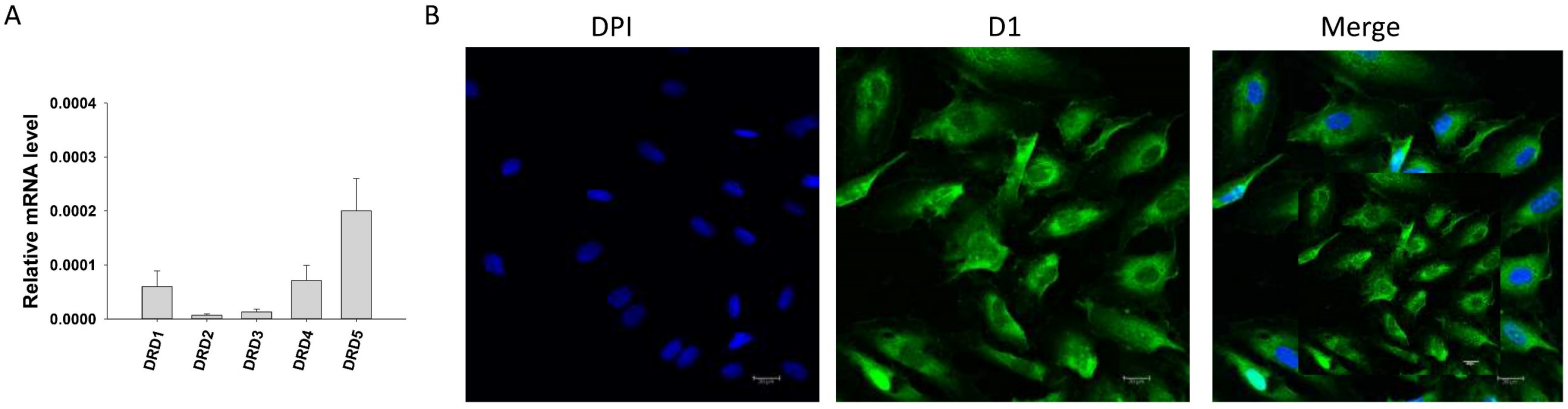
** Expression of dopamine receptors in HCMECs.** A. qPCR data showing the expression level (normalized to GAPDH) of D1- (*DRD1*), D2- (*DRD2*), D3- (*DRD3*), D4- (*DRD4*) and D5- (*DRD5*) dopamine receptors in HCMECs, n=5. B. Representative immunostaining with D1 dopamine receptor antibody showing expression of D1 receptor protein in HCMECs.

First, qPCR analysis was performed to examine the expression profile of dopamine receptors and some functional proteins in endothelial cells. Dopamine receptor D1 (*DRD1*), D2 (*DRD2*), D3 (*DRD3*), D4 (*DRD4*) and D5 (*DRD5*) (Figure 1 A), calcium-activated potassium channels including small conductance calcium-activated potassium channel type 1-3 (*KCNN1, KCNN2, KCNN3,* coding SK1, SK2 and SK3 channel, respectively), intermediate conductance calcium-activated potassium channel (*KCNN4*, coding SK4 channel) and big conductance calcium-activated potassium channel (*KCNMA1*, coding BKCa channel) (Figure S1A), as well as expression of nitric oxide synthase 3 (*NOS3*), NADPH oxidase 1 (*NOX1*), NADPH oxidase 2 (*NOX2*) and NADPH oxidase 5 (*NOX5*) (Figure S1B) were detected by qPCR. Immunostaining confirmed the existence of D1 (Figure 1B) and D2 (Figure S1C) receptor protein in HCMECs.

Then, patch clamp experiments were carried out to examine ion channel currents in HCMECs. Small conductance calcium-activated potassium channel currents (I_SK1-3_) were separated from other channel current by apamin, which is a blocker specific for SK1, SK2 and SK3 channels (Figure S2 A-C). If Ca2+-free solution was applied in the recording pipette, no apamin-sensitive current was detected (Figure S2 D-F), confirming existence of currents conducted by SK1-3 channels (I_SK1-3_).

**Figure 2:**
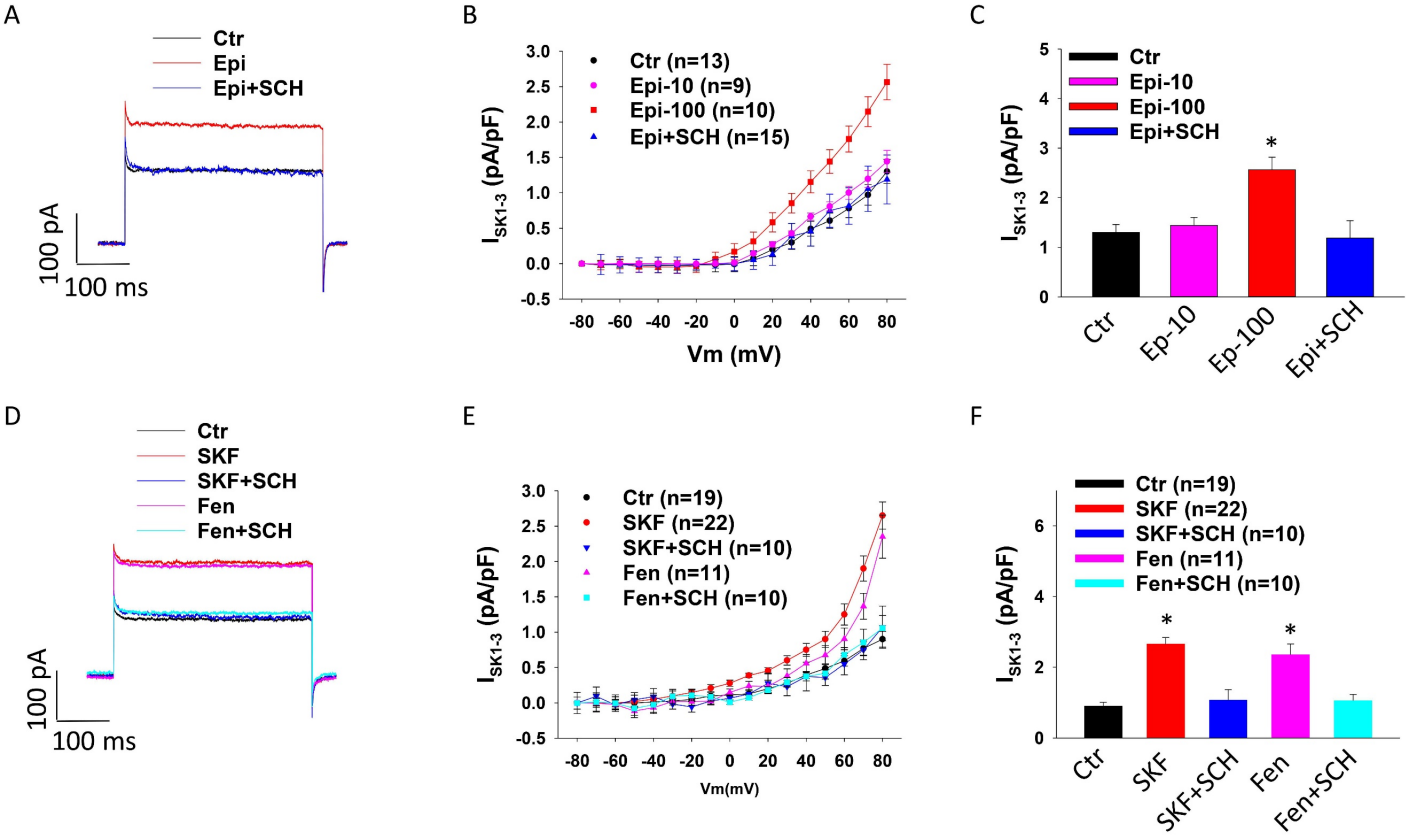
** D1/D5 receptor activation enhanced I_SK1-3_ in HCMECs.** I_SK1-3_ (apamin-sensitive currents) was isolated by apamin (1 μM), a specific blocker for SK1-3 channels. A. Representative traces of I_SK1-3_ at +80 mV (holding potential is -40 mV) in absence (Ctr) and presence of epinephrine (Epi,100 μM for 1 h) and epinephrine plus 10 μM SCH23390 (Epi+SCH). B. Current-voltage relationship (I-V) curves of I_SK1-3_ in absence (Ctr) and presence of 10 μM epinephrine for 1 h (Epi-10) and 100 μM epinephrine for 1 h (Epi-100) and 100 μM epinephrine plus 10 μM SCH23390 (Epi+SCH). C. Mean values of I_SK1-3_ at +80 mV in absence (Ctr) and presence of 10 μM epinephrine for 1 h (Epi-10) and 100 μM epinephrine for 1 h (Epi-100) and 100 μM epinephrine plus 10 μM SCH23390 (Epi+SCH). D. Representative traces of I_SK1-3_ at +80 mV in absence (Ctr) or presence of SKF38393 (SKF, 10 μM for 1 h) or fenoldopam (Fen, 5 μM for 1 h) or SKF38393 plus SCH23390 (SKF+SCH) or fenoldopam plus SCH23390 (Fen+SCH). E. I-V curves of I_SK1-3_ in each group. F. Mean values of I_SK1-3_ at +80 mV in each group. The n numbers represent number of measured cells. *p<0.05 versus Ctr determined by one-way ANOVA with Holm-Sidak post-test.

To check whether dopamine receptor activation contributes to toxic effects of catecholamine on I_SK1-3_, 10 µM or 100 µM epinephrine (Epi) was added to HCMECs to mimic the setting of TTC (catecholamine excess). Cells treated with 100 µM (but not 10 µM) Epi showed larger I_SK1-3_ and the high concentration Epi effect was significantly reduced by D1/D5 receptor blocker SCH23390 (Figure 2 A-C), suggesting an involvement of D1/D5 receptor activation in the Epi-effect on I_SK1-3_. qPCR analysis showed that Epi significantly enhanced SK2 expression but the effect was not significantly attenuated by SCH23390 (Figure S3 A). Western blot analysis detected no effect of Epi on SK2 expression (Figure S3 B-C). To confirm the contribution D1/D5 receptors to Epi effect on I_SK1-3_, two D1/D5-specific agonists, SKF38393 and fenoldopam, were applied. The results showed that SKF38393 and fenoldopam mimicked Epi-effect on I_SK1-3_ and SCH23390 abolished their effect (Figure 2 D-F), indicating that D1/D5 receptor activation enhanced SK channel activity.

### Protein kinase A is involved in the D1/D5-induced activation on SK channels

**Figure 3:**
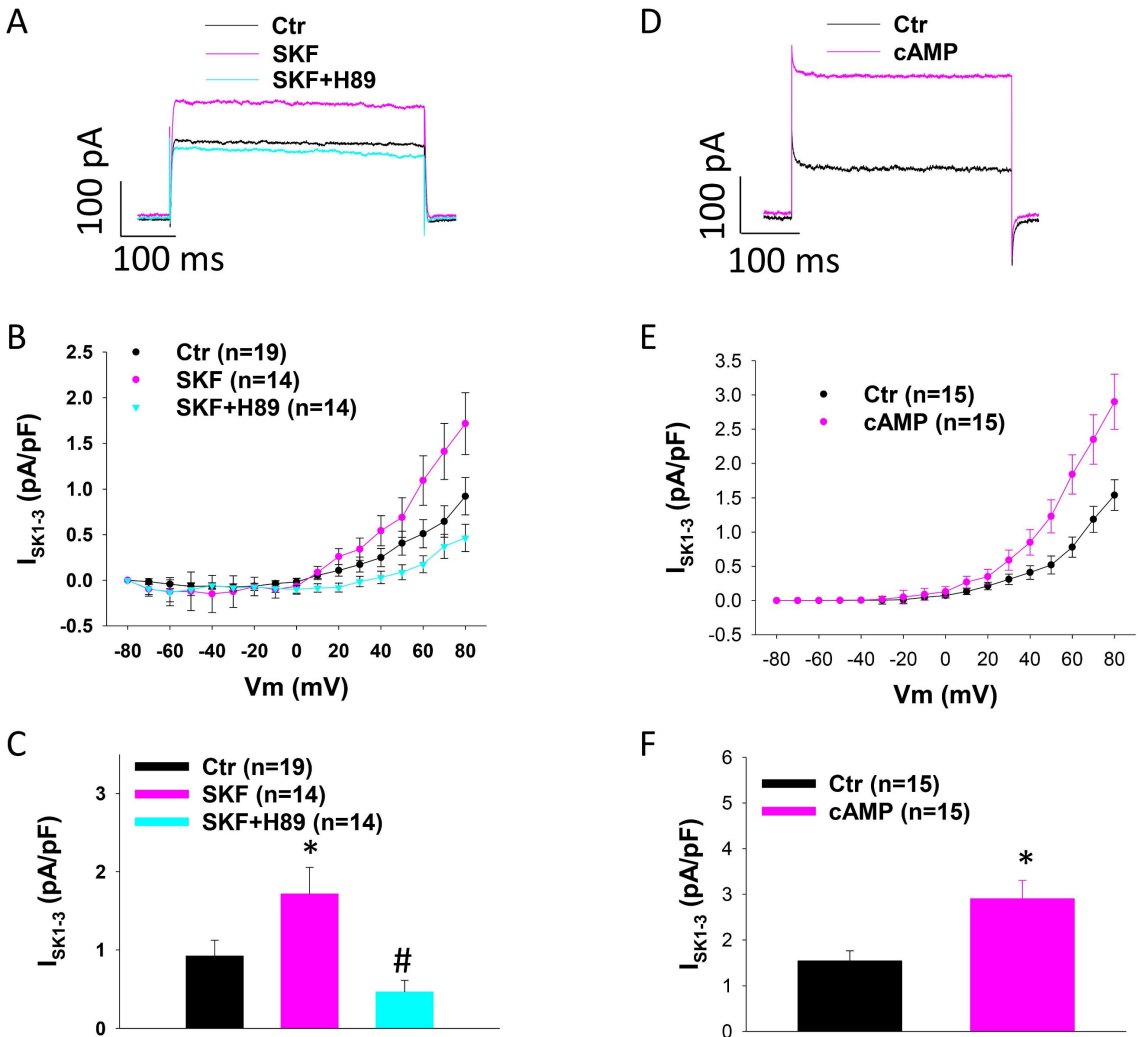
** Protein kinase A is involved in SKF38393 effect on SK1-3 channel current in HCMECs.** A. Representative traces of I_SK1-3_ at +80 mV (holding potential is -40 mV) in absence (Ctr) and presence of SKF38393 (SKF, 10 μM for 1 h) and SKF38393 plus 10 μM H89, a PKA inhibitor (SKF+H89). B. Current-voltage relationship (I-V) curves of I_SK1-3_ in each group. C. Mean values of I_SK1-3_ at +80 mV in each group. D. Representative traces of I_SK1-3_ at +80 mV in absence (Ctr) or presence of Sp-8-Br-cAMPS (cAMP, 10 μM for 1 h). E. I-V curves of I_SK1-3_ in each group. F. Mean values of I_SK1-3_ at +80 mV in each group. The n numbers represent number of measured cells. *p<0.05 versus Ctr, #p<0.05 versus SKF determined by one-way ANOVA with Holm-Sidak post-test (C) or unpaired t-test (F).

Since D1/D5-receptor is known to couple to Gs-cAMP-PKA signaling, we examined the involvement of PKA in SKF38393 effect on SK1-3 currents in HCMECs. For this, A PKA-blocker H89 and PKA activator Sp-8-Br-cAMPS (cAMP) were tested. Indeed, H89 abolished the effect of SKF38393 on I_SK1-3_ (Figure 3 A-C). cAMP mimicked the effect of SKF38393 on I_SK_ (Figure 3 D-F). These data indicate that PKA mediated D1/D5 receptor effect on SK channels.

### ROS mediated SKF effects on SK currents in HCMECs

**Figure 4:**
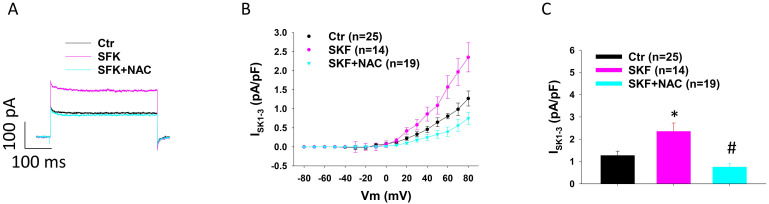
** ROS contributes to SKF38393 effect on I_SK1-3_ in HCMECs.** A. Representative traces of I_SK1-3_ at +80 mV in absence (Ctr) and presence of SKF38393 (SKF, 10 μM for 1 h) and SKF38393 plus 1 mM NAC (SKF+NAC). B. Current-voltage relationship (I-V) curves of I_SK1-3_ in each group. C. Mean values of I_SK1-3_ at +80 mV in each group. The n numbers represent number of measured cells. *p<0.05 versus Ctr, #p<0.05 versus SKF determined by one-way ANOVA with Holm-Sidak post-test.

It was reported that ROS production can contribute to the toxic effects of catecholamine excess on ion channel functions in cardiomyocytes 39, 40. Therefore, we tested the involvement of ROS in the increased I_SK1-3_ in HCMECs caused by D1/D5 receptor agonist. NAC (N-acetyl-l-cysteine), a ROS blocker, suppressed the effect of D1/D5 agonist (SKF38393) on I_SK1-3_ (Figure 4 A-C). H_2_O_2_ (a main form of endogenous ROS) mimicked SKF38393 effect on I_SK1-3_ (Figure S4).

### PKA is upstream of ROS

**Figure 5:**
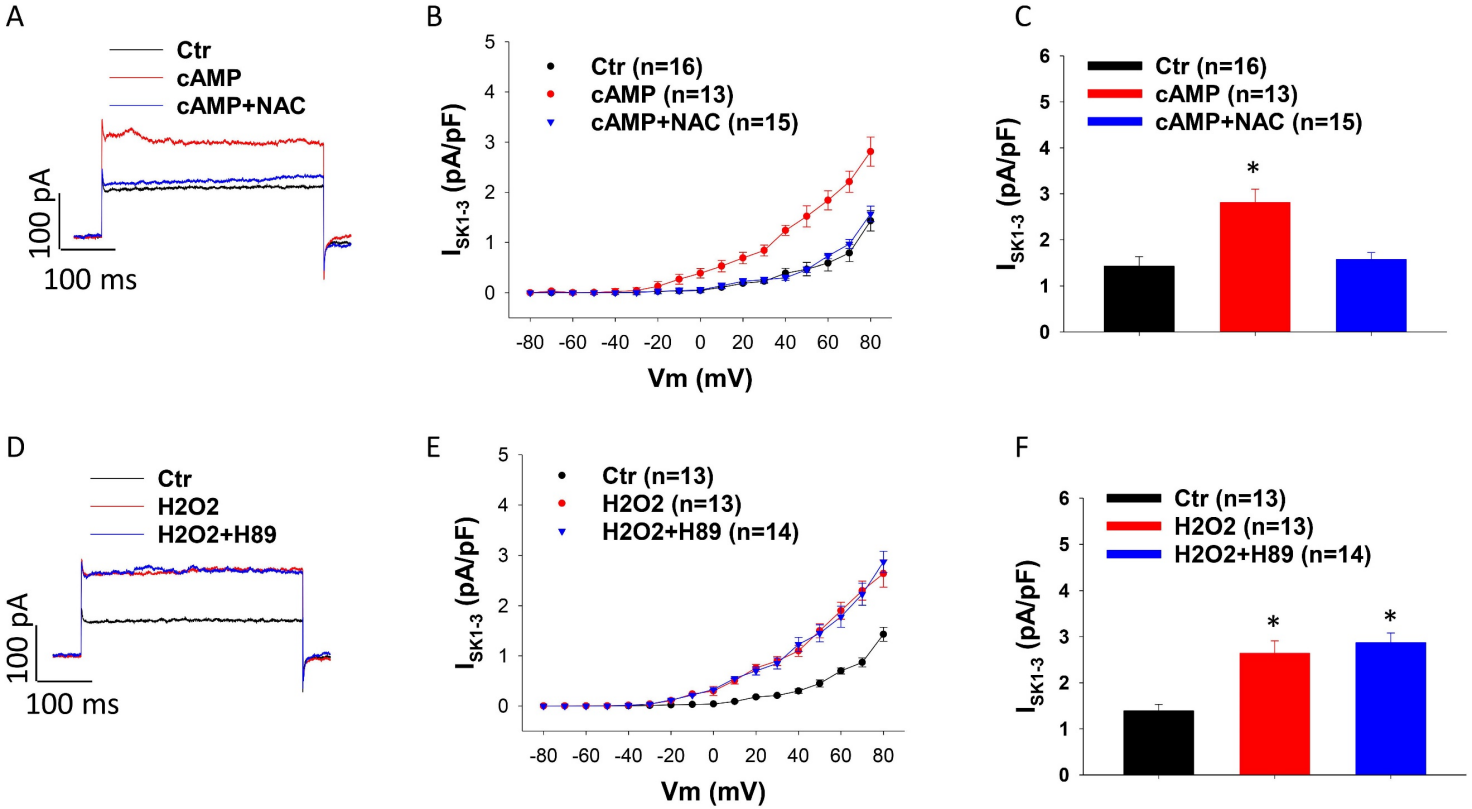
** ROS is downstream of PKA in D1/D5 receptor signaling.** A. Representative traces of I_SK1-3_ at +80 mV in absence (Ctr) and presence of Sp-8-Br-cAMPS (cAMP, 10 μM for 1 h) and cAMP plus 1 mM NAC (cAMP+NAC). B. Current-voltage relationship (I-V) curves of I_SK1-3_ in each group. C. Mean values of I_SK1-3_ at +80 mV in each group. D. Representative traces of I_SK1-3_ at +80 mV in absence (Ctr) or presence of hydrogen peroxide (H2O2, 100 μM for 1 h) and H2O2 plus 10 μM H89 (H2O2+H89). E. I-V curves of I_SK1-3_ in each group. F. Mean values of I_SK1-3_ at +80 mV in each group. The n numbers represent number of measured cells. *p<0.05 versus Ctr determined by one-way ANOVA with Holm-Sidak post-test.

Considering that both PKA and ROS were involved in effects of D1/D5 receptor on I_SK1-3_, we examined possible relationship of PKA and ROS in the signaling pathway. The PKA-inhibitor H89 was added to HCMECs 30 min before H_2_O_2,_ and in another group the ROS blocker NAC was added 30 min before cAMP. The results exhibited that NAC blocked the cAMP effect (Figure 5 A-C) but H89 failed to prevent the effect of H_2_O_2_ on I_SK1-3_ (Figure 5 D-F), suggesting that PKA is an upstream factor of ROS in the signaling pathway when D1/D5-receptor is activated.

### NADPH oxidase is involved in SKF effects on SK currents in HCMECs

**Figure 6:**
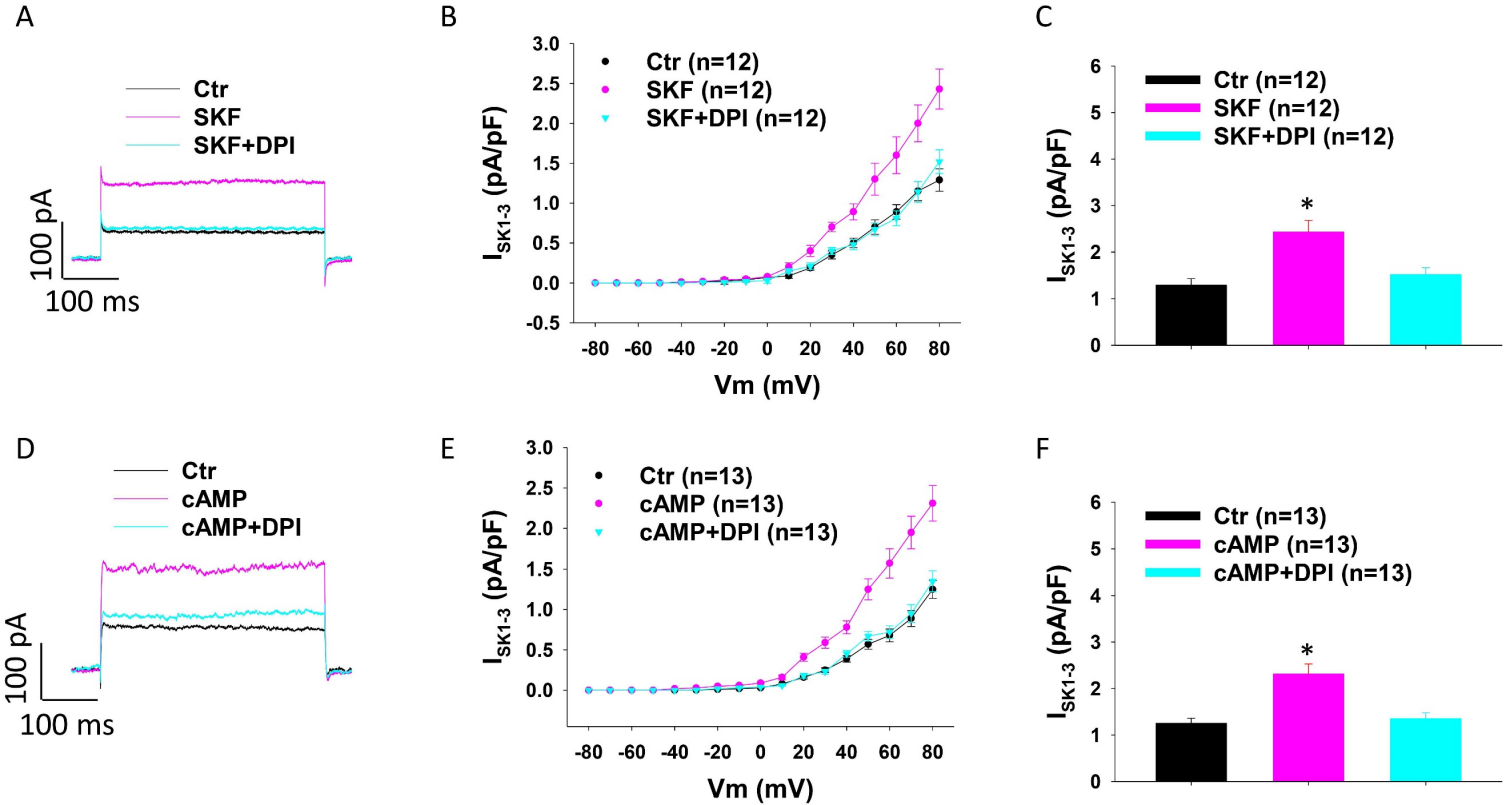
** NADPH oxidases are involved in SKF38393 and cAMP effects on I_SK1-3_ in HCMECs.** A. Representative traces of I_SK1-3_ at +80 mV in absence (Ctr) and presence of SKF38393 (SKF, 10 μM for 1 h) and SKF plus 10 μM NADPH oxidase inhibitor DPI (SKF+DPI). B. Current-voltage relationship (I-V) curves of I_SK1-3_ in each group. C. Mean values of I_SK1-3_ at +80 mV in each group. D. Representative traces of I_SK1-3_ at +80 mV in absence (Ctr) and presence of Sp-8-Br-cAMPS (cAMP, 10 μM for 1 h) and cAMP plus DPI (cAMP+DPI). E. I-V curves of I_SK1-3_ in each group. F. Mean values of I_SK1-3_ at +80 mV in each group. The n numbers represent number of measured cells. *p<0.05 versus Ctr determined by one-way ANOVA with Holm-Sidak post-test.

Given that ROS production contributes to the effects of dopamine receptor activation, we tested the involvement of NADPH oxidase, which catalyses the production of ROS, in the enhancement of I_SK1-3_ in HCMECs. DPI (Diphenyleneiodonium chloride), an inhibitor of NADPH oxidase, attenuated the effect of SKF38393 (Figure 6 A-C) and cAMP (Figure 6 D-F) on I_SK1-3_, suggesting the contribution of NADPH oxidases to effects of D1-PKA signaling.

### Effects of D1/D51 receptor and SK channel activation on the membrane potential of HCMECs

**Figure 7:**
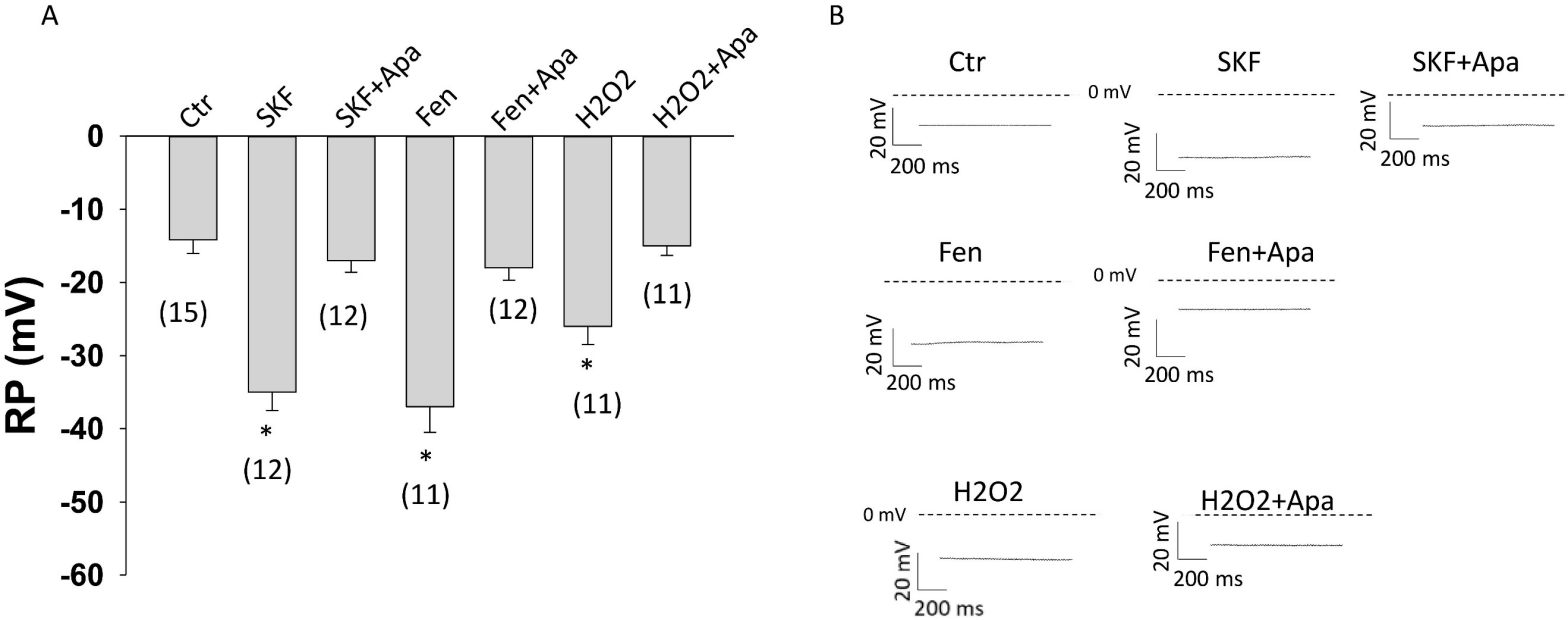
** Changes of cell membrane potential in HCMECs.** Cell membrane potentials were measured with patch clamp whole configuration (current clamp mode). A. Mean values of cell membrane potential (RP) in absence (Ctr) and presence of 10 μM SKF38393 (SKF), SKF 38393 plus 1 μM apamin (SKF+Apa), 5 μM fenoldopam (Fen), fenoldopam plus apamin (Fen+Apa), 100 μM hydrogen peroxide (H2O2) and H2O2 plus apamin (H2O2+Apa). B. Representative traces of RP in each group. The numbers in brackets represent number of measured cells. *p<0.05 versus Ctr determined by one-way ANOVA with Holm-Sidak post-test.

To examine whether D1/D5 receptor activation influences the membrane potential, the resting potential (RP) of HCMECs was analyzed. The D1/D5 agonists SKF38393 and fenoldopam caused a hyperpolarization (increased RP) (Figure 7 A-B). The SK1-3 channel blocker apamin reversed the effect of both agonists (Figure 7). Addition of 10 mM KCl in extracellular solution caused a depolarization and activation of SK1-3 channels by NS309 (activator of SK1-3 and SK4 channels) plus TRAM-34 (blocker of SK4) led to a hyperpolarization, but apamin alone only slightly reduced RP (Figure S5). These effects suggest that activation of SK1-3 channels by D1/D5 receptors can cause hyperpolarization of HCMECs. Furthermore, ROS (H2O2) could hyperpolarize the cell and apamin attenuated the effect (Figure 7), implying that ROS signaling can modulate endothelial membrane potential via SK1-3 channels.

### D1/D5 receptor and SK1-3 channels activation contributed to reduction of NO-production induced by epinephrine

**Figure 8:**
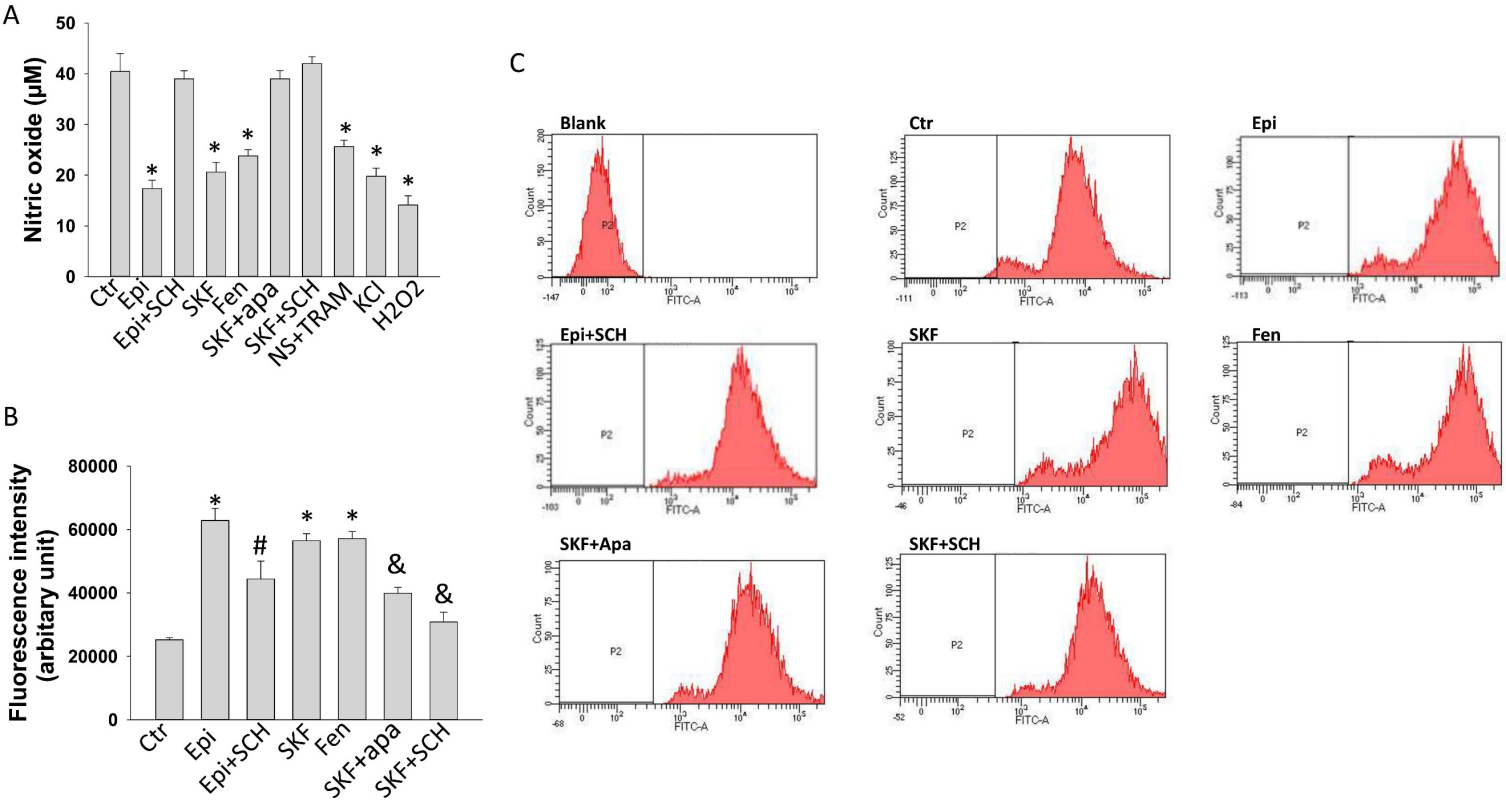
** Nitric oxide and ROS generation influenced by D1/D5 receptor and SK1-3 channels in HCMECs.** Nitric oxide (NO) concentration in culture medium of HCMECs was measured by ELISA and ROS level in HCMECs was measured by FACS. A. NO concentration in culture medium of HCMECs treated with vehicle (Ctr), 100 μM epinephrine (Epi), epinephrine plus 10 μM SCH23390 (Epi+SCH), 10 μM SKF38393 (SKF), 5 μM fenoldopam (Fen), SKF 38393 plus 1 μM apamin (SKF+Apa), SKF38393 plus SCH23390 (SKF+SCH),10 μM NS309+1 μM TRAM-34 (NS309+TR), 10 mM KCl (KCl) and100 μM H_2_O_2_ and (H_2_O_2_). B. ROS levels of HCMECs treated with vehicle (Ctr), 100 μM epinephrine (Epi), epinephrine plus 10 μM SCH23390 (Epi+SCH), 10 μM SKF38393 (SKF), 5 μM fenoldopam (Fen), SKF 38393 plus 1 μM apamin (SKF+Apa), SKF38393 plus SCH23390 (SKF+SCH). C. Representative FACS measurements showing ROS levels of HCMECs of each group. “Blank” means no ROS dye was applied. *p<0.05 versus Ctr, #p<0.05 versus Epi, &p<0.05 versus SKF determined by one-way ANOVA with Holm-Sidak post-test.

To explore possible roles of D1/D5 receptors in endothelial dysfunctions in the setting of TTC, the production of NO was measured in HCMECs treated with epinephrine and D1/D5 agonists SKF38393 or fenoldopam. Epinephrine, both SKF38393 and fenoldopam reduced NO production (Figure 8 A). The epinephrine effects were attenuated by SCH23390 and the SKF effect was abolished by apamin and SCH23390, indicating involvement of D1/D5 and SK1-3. Strikingly, both NS309 plus TRAM-34 and KCl reduced NO production (Figure 8A), excluding the possibility that activation of D1/D5 and SK1-3 channel suppress NO production via changing cell membrane potential. As expected, ROS (H2O2) reduced NO production (Figure 8A).

### D1/D5 receptor and SK1-3 channels activation contributed to increase in ROS production induced by epinephrine in HCMECs

Epinephrine and SKF38393 and fenoldopam enhanced ROS production (Figure 8 B-C). SCH23390 attenuated epinephrine and SKF38393 effect, apamin abolished the effects of SKF38393 (Figure 8 B-C), suggesting a participation of D1 receptor and SK1-3 channels in ROS production. Since SK channels were involved in changes of NO and ROS generation in presence of epinephrine and D1/D5 agonists and they contribute also to regulation of membrane potential, the influence of membrane potential on ROS generation was investigated. Actually, KCl decreased but NS309 plus TRAM-34 increased the production of ROS in HCMECs (Figure S6).

## Discussion

In the current study, the contribution of D1/D5 receptor and SK1-3 channel activation to catecholamine excess induced endothelial dysfunction and underlying mechanisms were investigated. The main novel findings in the study include 1) activation D1/D5 receptor can activate SK1-3 channels through cAMP-PKA signaling, 2) activation of SK1-3 channels caused by D1/D5 receptor can be mediated by NADPH-ROS signaling, 3) activation of D1/D5 receptor and SK1-3 channels can suppress NO production via increasing ROS rather than the cell membrane potential. These indicate that D1/D5 and SK1-3 channels may play important roles in catecholamine excess induced endothelial dysfunction.

Existence and functions of dopamine receptors in blood vessel have been reported 41, 42. The current study detected in HCMECs D1-D5 receptor gene expression by qPCR and D1, D2 protein expression by immunostaining. Furthermore, both agonist and antagonist of D1 receptor worked on endothelial cell function monitored by patch clamp (I_SK1-3_ and RP), ELISA (NO production) and FACS (ROS production). These demonstrate functional expression of dopamine receptors, at least D1, in HCMECs.

It has been reported that in bovine retina, besides dopamine, epinephrine could also activate dopamine D1 receptor 43. Another study showed that norepinephrine and epinephrine both could stimulate D4 receptor 44. Conversely, dopamine can also stimulate adrenoceptors 45, 46. These data may suggest an important cross-talk between adrenoceptors and dopamine receptors.

Previous studies and our recent study showed that dopamine or dopamine receptor may contribute to toxic catecholamine effects on cardiomyocytes in TTC 13, 25, 27, 47. However, whether dopamine receptor signaling contributes to endothelial dysfunction in TTC is so far unknown. The current study revealed that D1/D5 receptor activation (by agonists) can reduce NO production and increase ROS production, which may reflect endothelial dysfunction. Importantly, high concentration epinephrine exerted similar effects and D1/D5 blocker attenuated epinephrine effects. These support our hypothesis that in the setting of TTC high concentration catecholamine can activate dopamine receptor signaling, leading to endothelial dysfunction.

TTC-patients usually present typical changes in ECG such as elevation of ST-segment and inversion of T-wave, suggesting coronary spasm and myocardial hypoxia. Endothelial cells play important roles in controlling tonus of blood vessels and hence endothelial dysfunction may be a critical reason for coronary spasm in TTC. Indeed, it was reported that TTC-patients display endothelial dysfunctions monitored by flow-mediated vasodilatation measurements 33. The exact roles and mechanisms of endothelial dysfunction in TTC have not been clarified until now. The current study disclosed that catecholamine can suppress NO production by activating D1/D5 dopamine receptor signaling. NO is an important vasodilator, therefore, the reduction of NO generation, which can disrupt the balance between vasoconstrictors/vasodilators effects, may result in vasoconstriction. This may contribute to the coronary spasm in TTC.

It is well-know that EDHF (endothelial derived hyperpolarizing factor) besides NO and PGI2 (prostacyclin) plays also important roles for vascular tone 48-50. The exact molecular identity of EDHF is so far unclear, but nevertheless, some ion channels including calcium-activated potassium channels (mainly SK1-3 and SK4) and inward rectifier potassium channels (Kir) have been supposed to be candidates 50, 51. The second aim of the current study was to examine possible roles of SK1-3 channels in catecholamine-induced endothelial dysfunction. Two surprising findings were obtained in the current study: 1) epinephrine and D1/D5 receptor agonists enhanced I_SK-13_, 2) SK1-3 channel activation was involved in the reduction of NO production in presence of epinephrine or D1/D5 agonists. Increasing I_SK-13_ can hyperpolarize the cell and the hyperpolarization in endothelial cells can propagate into smooth muscle cells through myo-endothelial gap-junctions and in turn cause a relaxation of smooth muscle cells 35. On the other hand, the reduction of NO production, which should facilitate smooth muscle contraction, also contained the effect of SK1-3 channel activation (Figure 8A, SKF effect was reversed by apamin). How can we interpret the paradoxical effects occurring in HCMECs when D1/D5 receptor are activated?

To answer this critical question, further investigations were carried out. First, the effects of D1/D5 and SK1-3 channels on membrane potential were assessed. Then, the influence of membrane potential on NO generation was examined. It was observed that activation of D1/D5 and SK1-3 channels could cause a hyperpolarization of endothelial cells, but the NO production per se was membrane potential independent because both KCl (caused a depolarization) and NS309 plus TRAM-23 (caused a hyperpolarization) exerted similar effects (decreased NO generation). This suggests an extra mechanism underlying the reduction of NO production caused by D1/D5 and SK1-3 activation.

From literatures, it is known that ROS can reduce NO availability 52, 53. This led us to assess contribution of ROS to the reduction of NO production caused by D1/D5 and SK1-3 activation. Our experimental data showed that ROS (H2O2) reduced NO production, consistent with reported data. Here, the novel finding in our study is that D1/D5 and SK1-3 activation increased ROS production and the effect was probably mediated by the cell membrane hyperpolarization because a depolarization (caused by KCl) decreased but a hyperpolarization (caused by NS309 plus TRAM-34) increased ROS production. This may help understand how activation of SK1-3 channels reduced NO production in presence of dopamine receptor agonist, i.e., activation of D1/D5 receptor stimulates SK1-3 channels, which causes a hyperpolarization, the hyperpolarization enhances ROS generation, which suppresses NO generation. Of note, ROS could stimulate SK1-3 channels, and SK1-3 channel activation could increase ROS production, implying that a positive feedback modulation of ROS occurred. Since ROS can reduce NO availability, this positive feedback modulation of ROS may largely increase ROS production and strongly suppress NO generation, which may overcome the vasodilation effect of hyperpolarization, and therefor predominantly facilitate vasoconstriction.

The next question is how D1/D5 receptor activation stimulated SK1-3 channels. The increase in I_SK1-3_ can result from increase in either expression level or activity of channels. The former possibility was excluded by results that SCH23390 failed to influence epinephrine effect on SK channel expression. Although epinephrine increased SK2 expression at mRNA level, at protein level no effect was detected. It is very likely that D1/D5 activation enhanced I_SK1-3_ mainly by activating SK1-3 channels rather than increasing the channel expression level. To examine possible signaling responsible for SK1-3 activation in presence of D1 receptor agonists, different signaling blockers such as H89 (PKA inhibitor) and NAC (ROS blocker) as well as the signaling activators (cAMP, H2O2) were applied. From the effects of those substances, it can be speculated that PKA and ROS are involved in SK1-3 activation by D1/D5. Next, it could be illustrated that ROS is downstream of PKA because NAC could block cAMP effect but H89 failed to block H2O2 effect on SK1-3 channels. Further experiments exhibited that NADPH (nicotinamide adenine dinucleotide phosphate) oxidases contributed to effects of D1 receptor and PKA activators on SK1-3 channels. Taking data from this part of results together, it can be assumed that D1/D5 receptor activation activates SK1-3 channel through cAMP-PKA-NADPH-ROS signaling. Notably, a pathway of PKA-independent activation of NADPH-ROS cannot be excluded because DPI blocked not only cAMP effect but also SKF38393 effect.

ROS may come from different sources including mitochondria ROS and ROS produced by NADPH oxidases and other enzymes including xanthine oxidase, nitric oxide synthase, cyclooxygenases, cytochrome P450 enzymes and lipoxygenases 52. Our study suggests that D1/D5 activation can increase ROS generation through activating NADPH oxidases. Whether other sources of ROS can be influenced needs to explored in future study. ROS is an important vasoactivator and was reported to cause either vasoconstriction or vasodilation 29. It could be possible that high level ROS can cause a vasoconstriction through suppressing NO and other mechanisms.

In summary, epinephrine and D1/D5 receptor activators can stimulate SK1-3 channels via PKA and ROS signaling, leading to endothelial cell hyperpolarization. The hyperpolarization can enhance production of ROS, which can suppress NO generation. D1/D5 can activate NADPH oxidases through PKA-dependent and probably also PKA-independent pathway and increase the ROS generation. The study demonstrates that high level catecholamine may cause endothelial dysfunction through D1/D5-PKA-SK/ROS-NO signaling in the setting of TTC.

### Study limitations

Dopamine receptor family consists of two groups, D1/D5 and D2-4. This study did not investigate roles of D2-4 receptor although D2 was detected in HCMECs. Given that D1-like receptor blocker completely abolished Epi effects, D2-like receptors are coupled to Gi and cAMP mimicked effects of epinephrine and SKF on I_SK1-3_, it is unlikely that D2-like receptors contributed epinephrine effects.

Although our data showed that D1/D5 and PKA activation may enhance ROS generation through NADPH oxidases, but how NADPH oxidases were activated by PKA or D1/D5 receptor was not explored. Whether ROS directly activates SK1-3 or instead activates other proteins that regulate SK1-3 channels was not investigated.

Other channels besides SK1-3 may play important roles for endothelial functions. Whether dopamine receptor signalling can affect other channels needs to be investigated in future.

The study was performed at single cell level. Cell to cell interactions and *in vivo* conditions were not considered.

## Supplementary Material

Supplementary materials and methods, figures.
