# Supplementary material for: Roles and Mechanisms of Dopamine Receptor Signaling in Catecholamine Excess Induced Endothelial Dysfunctions
Source: Int J Med Sci. 2024 Jul 22;21(10):1964–75. doi: 10.7150/ijms.96550 (PMC11302566; doi:10.7150/ijms.96550)
Supplement: Supplementary file 1 — Supplementary materials and methods, figures. [file ijmsv21p1964s1.pdf]

Supplemental information

Roles and Mechanisms of Dopamine Receptor Signaling in Catecholamine Excess Induced Endothelial Dysfunctions

Running title: Dopamine receptor for endothelial dysfunction

Zhen Yang[1,2], Yingrui Li[2], Mengying Huang[2], Xin Li[2], Xuehui Fan[2,4], Chen Yan[2], Zenghui Meng[2], Bin Liao[5], Nazha Hamdani[6], Xiaoli Yang[1*], Xiaobo Zhou[2,3,4*], Ibrahim El-Battrawy[6,7] and Ibrahim Akin[2,3].

[1]Department of Ophthalmology, Affiliated Hospital of North Sichuan Medical College, 637000 Nanchong, Sichuan, China.

[2]First Department of Medicine, Medical Faculty Mannheim, University Medical Centre Mannheim (UMM), Heidelberg University, 68167 Mannheim, Germany.

[3]European Center for AngioScience (ECAS), German Center for Cardiovascular Research (DZHK) partner site Heidelberg/ Mannheim, and Centre for Cardiovascular Acute Medicine Mannheim (ZKAM), Medical Centre Mannheim, Heidelberg University, Germany.

[4]Key Laboratory of Medical Electrophysiology of Ministry of Education and Medical Electrophysiological Key Laboratory of Sichuan Province, Institute of Cardiovascular Research, Southwest Medical University, 646000 Sichuan, China.

[5]Department of Cardiac Macrovascular Surgery, Affiliated Hospital of Southwest Medical University, 646000 Sichuan, China

[6]Department of Cellular and Translational Physiology, Institute of Physiology, Ruhr University Bochum, 44801 Bochum, Germany and Institut für Forschung und Lehre (IFL), Molecular and Experimental Cardiology, Ruhr University Bochum, 44801 Bochum, Germany

[7]Bergmannsheil Bochum, Medical Clinic II, Department of Cardiology and Angiology, Ruhr University, Bochum, Germany

*Corresponding author

Address for correspondence:

1. Xiaoli Yang, MD

Department of Ophthalmology, Affiliated Hospital of North Sichuan Medical College, 637000 Nanchong, Sichuan, China. E-Mail: yangxiaolioculist@163.com

2. Xiaobo Zhou, MD

First Department of Medicine, Faculty of Medicine, University Medical Centre Mannheim, University of Heidelberg, Theodor-Kutzer-Ufer 1-3, 68167 Mannheim, Germany. Phone: 0049-621-383-1448. Fax: 0049-621-383-1474. **E-mail**: Xiaobo.zhou@medma.uni-heidelberg.de

Conflict of Interest: All authors declared no competing interests for this work.

Funding: This study was supported by German Center for Cardiovascular Research (DZHK) (81Z0500204 and 81X2500208), the project of co-operation of Nanchong City and North Sichuan Medical College (19SXHZ0075, 19SXHZ0067) and Sichuan Science and Technology Program (2022YFS0610).

Methods and materials

Ethics statement

The human cardiac microvascular endothelial cells (HCMECs) were bought from a company (PromoCell GmbH, Heidelberg, Germany, Cat.No:C-12285). The tissues used by PromoCell for the isolation of human cell cultures originate from donors who have signed a declaration of consent. PromoCell's donor acquisition, documents and cell collection process have been assessed and evaluated and approved by the ethical commission of the doctor's medical association of Baden-Württemberg (# 219-04).

Endothelial cell culture

HCMECs were cultured according to the instruction from the company. The cryopreserved cells were thawed and transferred to a cell culture flask containing the prewarmed medium with the composition: Endothelial Cell Growth Medium MV2 (C-22022, PromoCell, Heidelberg, Germany) supplemented with 0.05 ml/ml fetal bovine serum(FBS), 5 ng/ml recombinant human epidermal growth factor, 10 ng/ml recombinant human basic fibroblast growth factor, 20 ng/ml insulin-like growth factor, 0.5 ng/ml recombinant human vascular endothelial growth factor 165, 1 µg/ml ascorbic acid, 0.2 µg/ml hydrocortisone and 1% penicillin-streptomycin. The flask was placed in an

incubator (37°C, 5% CO_2) for 16-24 hours and the medium was replaced. Thereafter, the medium was changed every two days. The cells were split when they reached 80% confluency. After splitting, the cells (2 x 10^5/ml cells) were plated in new cell culture flasks with prewarmed Endothelial Cell Growth Medium MV2 and cultured in an incubator (37°C, 5% CO_2) with media change every two days. Cells of passage 3 to passage 7 were used for the study.

Polymerase chain reaction assays

Quantitative polymerase chain reaction assays (qPCR) were carried out according to the provided protocol. Total RNA in HCMECs was extracted with RNeasy mini kit (Total RNA extraction reagent) (Qiagen, Hilden, Germany). The extracted RNA (10 µl) was reverse transcribed into cDNA using High-Capacity cDNA Reverse Transcription Kit (Thermo Fisher) on a Stratagene MX 3005P Real-Time Cycler (Stratagene, USA). The cDNA synthesized from total RNA was amplified by the Real-time PCR System (Applied StepOnePlus Real-Time PCR Systems, Thermo Fisher) using a PCR mix with hot start Taq DNA polymerase and SYBR Green (Sibir Rox Hot Mastermix, Catnumber 119405, BIORON, Germany) in the presence of sense and antisense primers (400 nM each).

According to the threshold cycle (CT), the ΔΔCT method was used to calculate the mRNA expression of the target gene relative to the housekeeping gene GAPDH in a sample of treated or untreated (control) cells, as a fold change =

$2^{-\Delta\ (\Delta CT)}$, where ΔCT = CTgene of interest-CTGAPDH and Δ (ΔCT) = ΔCTtreated-ΔCTcontrol. For reducing technical variability, the control and treatment groups were repeated three times independently. From each experiment, cDNA was measured from at least three cell culture wells as biological replicates for each treatment. Each sample was added in two wells as technical replicates to repeat the measurements.

Western blot

The HCMECs were split from culture flask by ice-cold RIPA lysis buffer (Sigma, #r0278) with protease inhibitor (Sigma-Aldrich #P8340-1ML). BCA protein assay reagents (Thermo Fisher Scientific, #23227) were used to measure protein concentration. 20 µg protein separated by sodium dodecyl sulfate polyacrylamide gel electrophoresis (SDS-PAGE) was electro-transferred to polyvinylidene difluoride (PVDF) membranes by semi-dry electrophoretic transfer cell (Bio-Rad). After, membranes were blocked at room temperature for 1 hour with 5% nonfat dry milk powder, which was dissolved in Tris-buffered saline with 0.1% Tween-20 (TBST), primary antibodies were used to incubated membranes (4°C overnight). On the next day, peroxidase-conjugated secondary antibodies that were dissolved in TBST were applied to incubate membranes at room temperature for 1 hour. Immunoreactive bands on the membrane were detected by enhanced chemiluminescence (Thermo Fisher Scientific, #1859697 and #1859700) by autoradiography. SK2 (Alomone, APC-

028) and GAPDH (HyTest #5G4) primary antibodies as well as GAR (Sigma Aldrich #A0545) and GAM (Sigma Aldrich #A3682) secondary antibodies were used.

Immunofluorescence (IF) staining

HCMECs were incubated at 37 °C with 0.05% Trypsin-EDTA (Life Technologies) for 2 to 4 min. After RPMI medium containing 10% FBS was added, cells were centrifuged with 250 × g at room temperature for 4 min. After the supernatant was discarded, the cells were resuspended in basic culture medium and pipetted onto culture slides (FALCON 354114). Slides stayed overnight. After they were washed 2 to 3 times with PBS, they were fixed with 4% paraformaldehyde (Sigma) for 20 min at room temperature. Next, cells were washed with PBS 2 to 3 times and then permeabilized with 0.1% Triton-X100 (Carl Roth) for 10 min. Thereafter, they were washed 2 to 3 times with PBS and blocked with 5% bovine serum albumin (BSA; Sigma-Aldrich) in PBS for 1 h at 4°C. Primary antibodies were utilized in 5% BSA overnight at 4°C. Then, cells were washed with PBS and incubated for 1 h at RT with corresponding secondary antibodies conjugated to Alexa Fluor 488 or 642 (1:200). Next, cells were washed with PBS and incubated with DAPI (Biozol) for 10 min at room temperature in the dark. Images were obtained by the Confocal Microscope TCS SP-8 upright (Leica, Germany) with Plan-Apochromat 40×/0.6 objective.

Patch-clamp

The HCMECs cultured for 2-4 hours after passaging were utilized for patch clamp measurements. The whole-cell patch-clamp recording technique was utilized for recording ion channel currents and resting potential (RP) of cells. The SK1-3 channel specific blocker apamin was applied for separating current of SK1-3 channels from other types of ion channels. The apamin-sensitive current was used for the analyses of SK1-3 channel currents. For minimizing the influence of the rundown of recorded currents on the experimental results, the recordings were started after the current reached a stable state, normally 3 to 5 minutes after the whole cell configuration was established. The patch electrode was pulled from borosilicate glass capillary (MTW 150F; World Precision Instruments, Sarasota, FL) by the DMZ universal puller (Zeitz-Instrumente Vertriebs GmbH, Martin Reed, Germany). The pipette resistance was 2 to 3 MΩ and 4 to 5 MΩ for current and RP measurements, respectively.

After the patch pipette was carefully moved on the cell membrane by a micromanipulator, a slight suction (negative pressure) was usually needed for obtaining a Giga-Ohm seal between cell membrane and the pipette wall. After the Giga-Ohm seal was formed, membrane that was sucked into the pipette was raptured by a stronger suction to obtain a whole cell configuration. Signals were acquired at 10 kHz, after being filtered at 2 kHz, with the EPC10 Patch-master digitizer hardware (HEKA Germany) and Fit-master software (HEKA

Germany). All the measured ion channel currents were normalized to the membrane capacitance for obtaining current densities in pA/pF. Current densities were plotted against the respective voltages for obtaining current-voltage (I-V) relationship curves.

The resting potential (RP) was measured in current-clamp mode (CC-mode). Since RP decreased quickly after the cell membrane in the pipette was ruptured, the RP was measured immediately (within 1 min) after the whole cell configuration was established.

The extracellular (bath) solution for SK1-3 channel currents and RP measurements comprised 127 mmol/L NaCl, 5.9 mmol/L KCl, 2.4 mmol/L $CaCl_2$, 1.2 mmol/L $MgCl_2$, 11 mmol/L glucose, and 10 mmol/L HEPES. The pH value was adjusted to 7.4 with NaOH. The intracellular (pipette) solution contained 6 mmol/L NaCl, 126 mmol/L KCl, 1.2 mmol/L $MgCl_2$, 5 mmol/L EGTA, 11 mmol/L glucose, 10 mmol/L HEPES and 1 mmol/L Mg-ATP. The pH value was adjusted to 7.2 with KOH. For obtaining the free Ca^{2+} concentration of 0.5 μM, appropriate $CaCl_2$ was added according to the calculation by the software MAXCHELATOR (http://web.stanford.edu/~cpatton/downloads.htm).

Measurement of nitric oxide production by endothelial cells

The nitric oxide production in HCMECs culture supernatants was measured by ELISA using a Nitric Oxide Assay Kit (Thermo Fisher). The kit contains the enzyme Nitrate Reductase for converting nitrate to nitrite. Nitrite is measured as a colored azo dye product of the Griess reaction that absorbs visible light at

540 nm. The interaction of NO was determined by both nitrate and nitrite concentrations in the culture supernatants. All standards and samples were run in duplicate. Nitrite assay and nitrate assay were performed following the protocols provided by the company.

The measured data were analyzed with a 4-parameter logistic curve-fitting program. The concentration of nitrite or total nitrate were calculated by the following method:

1. The average net optical density (OD) bound for each standard and sample was calculated by subtracting the average zero standard OD from the average OD for each standard and sample. Average Net OD = Average OD - Average Zero Standard OD.

2. The average net OD for each standard was plotted versus nitrite or nitrate concentration.

3. The average OD for each sample was plotted and nitrite or total nitrite and nitrate concentration were interpolated from the graph.

4. The nitrite concentration was subtracted from the total nitrite and nitrate concentration to obtain the nitrate concentration in the sample.

Measurement of ROS production

The reactive oxygen species (ROS) production in HCMECs was measured by FACS using the 2′,7′-Dichlorofluorescin diacetate (DCFH-DA, sigma) method with the ROS assay kit. DCFH-DA, a non-polar fluorescence probe that can penetrate cell membranes, is converted into DCFH in cells and then detected by flow cytometry. The HCMCs were plated into the 15ml tubes at a density of 1×10^6 cells/tubes and incubated with 10 μM DCFH-DA for 30 minutes at 37°C

in the dark. After the cells were washed 3 times with PBS, they were measured by BD FACSCanto™ II (Becton Dickinson, Heidelberg, Germany). Analysis was performed with a quantitative method via BD FACS Diva software (Version 8.0.1). The fluorescence was measured at 488 nm (excitation) and at 530nm (emission). At least 25,000 events were acquired and analyzed per experimental condition.

Statistics

All data are shown as mean ± SEM and were analyzed with the software SigmaPlot 14.0 (Systat GmbH, Germany). The unpaired t-test was used for comparisons of two independent groups with normal distribution. One-way ANOVA with Holm-Sidak post-test was performed for comparisons of more than two groups. $P < 0.05$ (two-tailed) was considered significant.

Figures

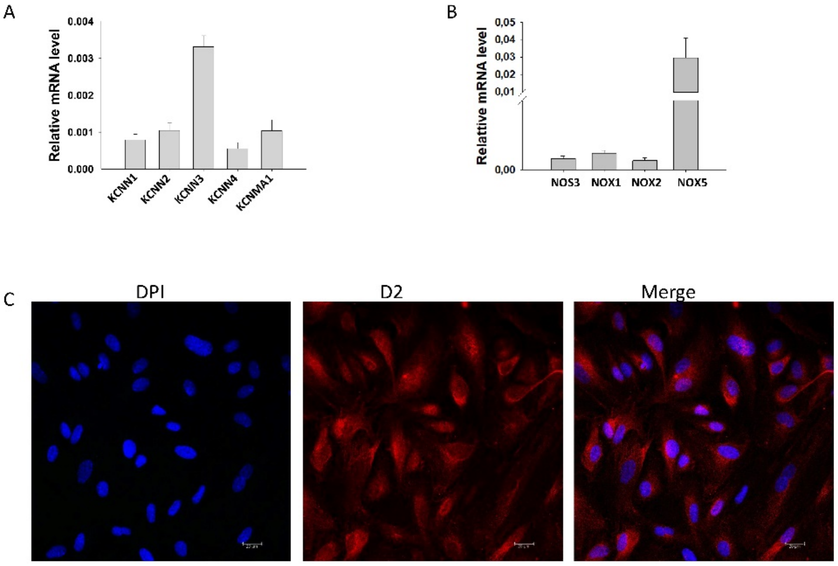
A
Relative mRNA level
0.004
0.003
0.002
0.001
0.000
KCNN1
KCNN2
KCNN3
KCNN4
KCNMA1
B
Relative mRNA level
0,05
0,04
0,03
0,02
0,01
0,00
NOS3
NOX1
NOX2
NOX5
C
DPI
D2
Merge

Fig. S1 Expression of ion channels, D2 dopamine receptor, nitric oxide synthase and NADPH oxidase in HCMECs. A. qPCR data showing the expression level of small conductance calcium-activated potassium channel type 1(*KCNN1*, SK1), type 2 (*KCNN2*, SK2), type 3 (*KCNN3*, SK3) and type 4 (*KCNN4*, SK4) as well as big conductance calcium-activated potassium channel (*KCNMA1*, BKCa) in HCMECs, n=5. B. qPCR data showing the expression level of nitric oxide synthase 3 (NOS3), NADPH oxidase 1 (*NOX1*), NADPH oxidase 2 (*NOX2*) and NADPH oxidase 5 (*NOX5*) in HCMECs, n=5. C. Representative immunostaining with D2 dopamine receptor antibody showing expression of D2 receptor protein in HCMECs.

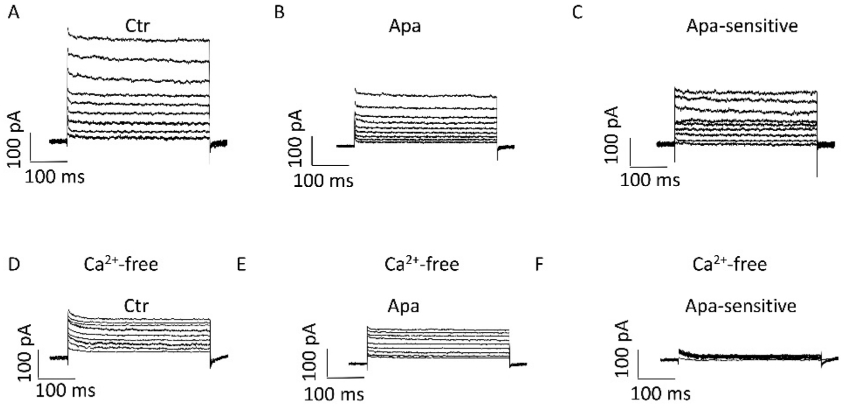

Fig. S2 Small conductance calcium-activated potassium channel currents in HCMECs. A. Representative traces of membrane current from 0 to +80 mV (holding potential is -40 mV) before application of apamin. B. Representative traces of membrane current from 0 to +80 mV after application of apamin. C. Apamin-sensitive current, which is defined as $I_{Sk1\text{-}3}$. In A-C, the intracellular free calcium concentration was 500 nM. D. Representative traces of membrane current from 0 to +80 mV recorded with calcium-free intracellular solution before application of apamin. E. Representative traces of membrane

current from 0 to +80 mV recorded with calcium-free intracellular solution after application of apamin. F. Apamin-sensitive currents recorded with calcium-free intracellular solution.

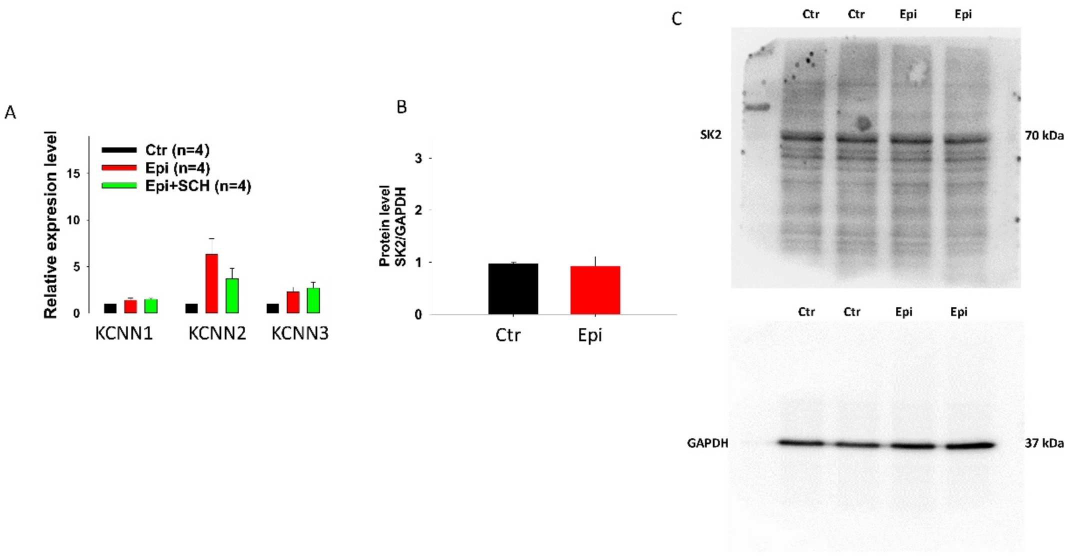

Fig. S3 SK channel expression in HCMECs challenged by epinephrine and dopamine receptor blocker. A. mRNA levels of SK1, SK2 and SK3 in HCMECs in absence (Ctr) or presence of epinephrine (Epi, 100 μM for 1 h) or epinephrine plus 10 μM SCH23390 (Epi+SCH). B. Mean values of western blot analyses showing protein levels of SK2 in HCMECs in absence (Ctr) or presence of epinephrine (Epi, 100 μM for 1 h). C. Representative bands of western blot showing protein levels of SK2 in HCMECs in absence (Ctr) or presence of epinephrine (Epi, 100 μM for 1 h). The n numbers present numbers of experiments. The n number in B is n=3.

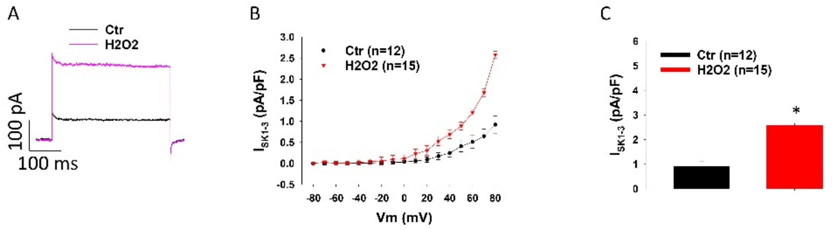

Fig. S4 ROS enhanced $I_{SK1\text{-}3}$ in HCMECs. A. Representative traces of $I_{SK1\text{-}3}$ at +80 mV in absence (Ctr) or presence of hydrogen peroxide (H2O2, 100 μM for 1 h). B. I-V curves of $I_{SK1\text{-}3}$ in each group. C. Mean values of $I_{SK1\text{-}3}$ at +80 mV in each group. The n numbers represent number of measured cells. *$p<0.05$ versus Ctr determined unpaired t-test.

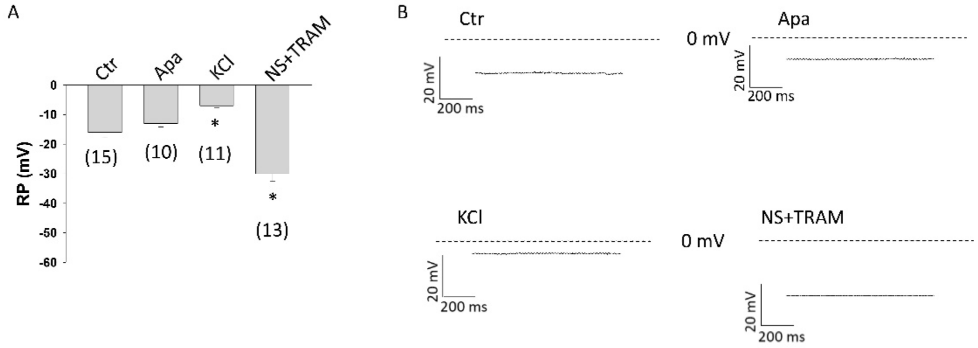

Fig. S5 Changes of cell membrane potential in HCMECs. Cell membrane potentials were measured with patch clamp whole configuration (current clamp mode). A. Mean values of cell membrane potential (RP) in absence (Ctr) and presence of 1 μM apamin (Apa), 10 mM KCl (KCl), 10 μM NS309+1 μM TRAM-34 (NS309+TR). B. Representative traces of RP in each group. The numbers in brackets represent number of measured cells. *p<0.05 versus Ctr determined by one-way ANOVA with Holm-Sidak post-test.

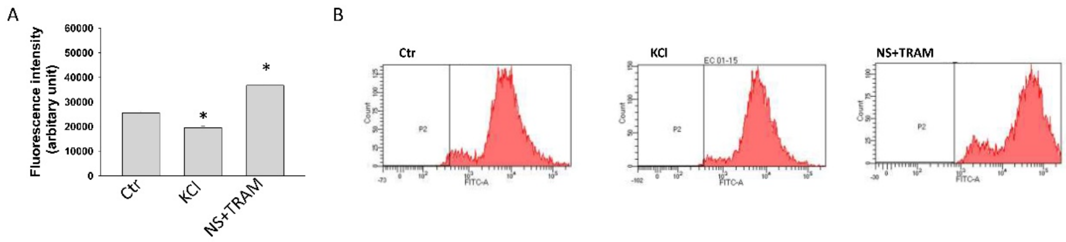

Fig. S6 ROS generation influenced by membrane potential in HCMECs. ROS level in HCMECs was measured by FACS. A. ROS levels of HCMECs treated with vehicle (Ctr) or 10 mM KCl (KCl) or 10 μM NS309+1 μM TRAM-34 (NS+TRAM). B. Representative FACS measurements showing ROS levels of HCMECs of each group. *$p<0.05$ versus Ctr determined by one-way ANOVA with Holm-Sidak post-test.
